# The safety and efficacy of umbilical cord blood mononuclear cells in individuals with spastic cerebral palsy: a randomized double-blind sham-controlled clinical trial

**DOI:** 10.1186/s12883-022-02636-y

**Published:** 2022-03-29

**Authors:** Morteza Zarrabi, Masood Ghahvechi Akbari, Man Amanat, Anahita Majmaa, Ali Reza Moaiedi, Hadi Montazerlotfelahi, Masoumeh Nouri, Amir Ali Hamidieh, Reza Shervin Badv, Hossein Karimi, Ali Rabbani, Ali Mohebbi, Shahram Rahimi-Dehgolan, Rosa Rahimi, Ensieh Dehghan, Massoud Vosough, Saeed Abroun, Farhad Mahvelati Shamsabadi, Ali Reza Tavasoli, Houman Alizadeh, Neda Pak, Gholam Reza Zamani, Mahmoud Mohammadi, Mohsen Javadzadeh, Mohammad Ghofrani, Seyed Hossein Hassanpour, Morteza Heidari, Mohammad Mehdi Taghdiri, Mohamad Javad Mohseni, Zahra Noparast, Safdar Masoomi, Mehrdad Goudarzi, Masood Mohamadpour, Razieh Shodjaee, Solaleh Samimi, Monireh Mohammad, Mona Gholami, Nahid Vafaei, Leyli Koochakzadeh, Amir Valizadeh, Reza Azizi Malamiri, Mahmoud Reza Ashrafi

**Affiliations:** 1 Department of Regenerative Medicine, Cell Science Research Center, Royan Institute for Stem Cell Biology and Technology, ACECR, Tehran, Iran; 2 Physical Medicine and Rehabilitation Department, Children’s Medical Center, Tehran University of Medical Sciences, Tehran, Iran; 3 Division of Neurogenetics and Neuroscience, The Moser Center for Leukodystrophies, Kennedy Krieger Institute, Johns Hopkins University, Baltimore, MD USA; 4 Pediatrics Center of Excellence, Pediatric Intensive Unit, Children’s Medical Center, Tehran University of Medical Sciences, Tehran, Iran; 5 Department of Pediatric Neurology, Clinical Research Development Center of Children Hospital, Hormozgan University of Medical Sciences, Bandar Abbas, Iran; 6 Department of Pediatrics, Faculty of Medicine, Tehran Medical Sciences, Islamic Azad University, Tehran, Iran; 7 R & D Department, Royan Stem Cell Technology Co, Tehran, Iran; 8 Pediatrics Center of Excellence Pediatric Hematology, Oncology and Stem Cell Transplantation Department, Children’s Medical Center, Tehran University of Medical Sciences, Tehran, Iran; 9 Pediatrics Center of Excellence, Department of Pediatric Neurology, Children’s Medical Center, Growth and Development Research Center, Tehran University of Medical Sciences, Tehran, Iran; 10 Neurorehabilitation Research Center University of Welfare and Rehabilitation Sciences, Tehran, Iran; 11 Pediatrics Center of Excellence Pediatric Endocrinology Department, Growth and Development Research Center, Children’s Medical Center, Tehran University of Medical Sciences, Tehran, Iran; 12 Pediatrics Center of Excellence, Growth and Development Research Center, Children’s Medical Center, Tehran University of Medical Sciences, Tehran, Iran; 13 Physical Medicine and Rehabilitation Department, Tehran University of Medical Sciences, Tehran, Iran; 14 Physical Medicine and Rehabilitation Department, Khatamolanbia Hospital, Tehran, Iran; 15 Transplantation Department, Royan Stem Cell Technology Co, Tehran, Iran; 16 Department of Hematology, Faculty of Medical Sciences, Tarbiat Modares University, Tehran, Iran; 17 Department of Pediatric Neurology, Atieh Hospital, Tehran, Iran; 18 Pediatrics Center of Excellence, Department of Radiology, Children’s Medical Center, Tehran University of Medical Sciences, Tehran, Iran; 19 Department of Pediatric Neurology, Mofid Children’s Hospital, Pediatric Neurology Research Center, Shahid Beheshti University of Medical Sciences, Tehran, Iran; 20 Department of Pediatric Neurology, Aliasghar Children’s Hospital, Iran University of Medical Sciences, Tehran, Iran; 21 Pediatric Urology Research Center, Pediatrics Center of Excellence, Children’s Medical Center, Tehran University of Medical Sciences, Tehran, Iran; 22 Department of Pediatric Nephrology, Bahrami Hospital, Tehran University of Medical Sciences, Tehran, Iran; 23 Department of Epidemiology and Biostatistics, School of Public Health, Tehran University of Medical Sciences, Tehran, Iran; 24 Department of Pediatric Anesthesiology, Children’s Medical Center, Tehran University of Medical Sciences, Tehran, Iran; 25 Process Department, Royan Stem Cell Technology Co, Tehran, Iran; 26 Faculty of Medicine, Students’ Scientific Research Center, Tehran University of Medical Sciences, Tehran, Iran; 27 Pediatrics Center of Excellence Pediatric Hematology, Department of Hematology & Oncology, Children’s Medical Center, Tehran University of Medical Sciences, Tehran, Iran; 28 Department of Paediatric Neurology, Golestan Medical, Educational, and Research Center, Ahvaz Jundishapur University of Medical Sciences, Ahvaz, Iran

**Keywords:** Cerebral palsy, Stem cells, Umbilical cord, Mononuclear cells, Children

## Abstract

**Introduction:**

The current multi-center, randomized, double-blind study was conducted among children with cerebral palsy (CP) to assess the safety and efficacy of umbilical cord blood mononuclear cell (UCB-MNC). We performed the diffusion tensor imaging to assess the changes in the white matter structure.

**Methods:**

Males and females aged 4 to 14 years old with spastic CP were included. Eligible participants were allocated in 4:1 ratio to be in the experimental or control groups; respectively. Individuals who were assigned in UCB-MNC group were tested for human leukocyte antigen (HLA) and fully-matched individuals were treated with UCB-MNCs. A single dose (5 × 10^6^ /kg) UCB-MNCs were administered via intrathecal route in experimental group. The changes in gross motor function measure (GMFM)-66 from baseline to one year after treatment were the primary endpoints. The mean changes in modified Ashworth scale (MAS), pediatric evaluation of disability inventory (PEDI), and CP quality of life (CP-QoL) were also evaluated and compared between groups. The mean changes in fractional anisotropy (FA) and mean diffusivity (MD) of corticospinal tract (CST) and posterior thalamic radiation (PTR) were the secondary endpoints. Adverse events were safety endpoint.

**Results:**

There were 72 included individuals (36 cases in each group). The mean GMFM-66 scores increased in experimental group; compared to baseline (+ 9.62; 95%CI: 6.75, 12.49) and control arm (β: 7.10; 95%CI: 2.08, 12.76; Cohen’s d: 0.62) and mean MAS reduced in individuals treated with UCB-MNCs compared to the baseline (-0.87; 95%CI: -1.2, -0.54) and control group (β: -0.58; 95%CI: -1.18, -0.11; Cohen’s d: 0.36). The mean PEDI scores and mean CP-QoL scores in two domains were higher in the experimental group compared to the control. The imaging data indicated that mean FA increased and MD decreased in participants of UCB-MNC group indicating improvements in white matter structure. Lower back pain, headaches, and irritability were the most common adverse events within 24 h of treatment that were related to lumbar puncture. No side effects were observed during follow-up.

**Conclusions:**

This trial showed that intrathecal injection of UCB-MNCs were safe and effective in children with CP.

**Trial Registration:**

The study was registered with ClinicalTrials.gov (NCT03795974).

**Supplementary Information:**

The online version contains supplementary material available at 10.1186/s12883-022-02636-y.

## Introduction

Cerebral palsy (CP) is a heterogeneous group of permanent neuro-developmental disorders that affects the muscle tone, movements, and motor skills [1]. CP is the leading cause of childhood disability and the global prevalence was reported to be about 3 per 1000 live births [2]. Individuals with CP have higher prevalence of high-burden medical events and higher economic burden compared to the general population [3, 4]. Early therapy may reduce the burden of CP. To date, no disease-modifying treatments have been found in CP and the current therapies are focused on treating disabilities and managing associated co-morbidities. The integral treatments of this condition includes physical therapy and rehabilitations but have limited efficacy in most cases.

Stem cell therapy was shown to be a promising treatment in various neurological disorders; including stroke, amyotrophic lateral sclerosis, Parkinson’s disease, and spinal cord injury [5–8]. Some randomized clinical trials reported that stem cell therapy can offer the potential to treat CP [9–12]. There are several types of stem cells that can be extracted from different sources. The umbilical cord blood (UCB) has been used for years to treat leukemia and anemias [13]. The mononuclear cells (MNCs) derived from UCB contain hematopoietic lineage cells; including lymphocytes, monocytes, stem cells, and endothelial progenitor cells as well as mesenchymal stromal cells (MSCs). Some studies assessed the therapeutic effects of the MNCs isolated from the bone marrow but cord blood administration is generally more convenient in pediatric population as cells can be provided without invasive and painful procedures. The use of allogenic umbilical cord blood is also cheaper and less time-consuming than the use of autologous bone marrow cells. The allogenic UCB-MNCs were intravenously administered in recent phase II clinical studies and showed to be safe and effective [14, 15]. It was noted that fully human leukocyte antigen (HLA)-matched and 1-mismatched cord blood units administered to the subjects yielded better motor outcomes than those in the 2-mismatched group [15].

This randomized double-blind sham-controlled clinical study further evaluated the safety and efficacy of the UCB-MNCs in the treatment of children and adolescents with CP. We assessed if single intrathecal administration of 6/6 HLA-matched UCB-MNCs in patients could improve clinical and imaging outcomes. Prior studies showed that small proportion of cells that infused via intravenous route were likely to traverse the pulmonary microvasculature and reach the arterial circulation [16]. Here, we injected single and lower dose of cells using intrathecal route to assess its therapeutic effects in improvements of gross motor function, spasticity, disability score, and quality of life. The quantitative diffusion tensor imaging (DTI) was conducted in all individuals before and after treatment to more rigorously assess the efficacy of cell-based therapy. We hypothesized that the clinical and imaging endpoints could be significantly improved in participants treated with the UCB-MNCs compared to the sham-control arm (superiority trial).

## Methods

### Study design and eligibility criteria

This was a multi-center, randomized, double-blind, population-based clinical study with sham-control group conducted in Children’s Medical Center affiliated to Tehran University of Medical Sciences, with the assistance of Bandar Abbas pediatric hospital in Hormozgan province. The patients referred from different provinces of Iran to our medical center. The Bobath concept was used as the rehabilitation approach in all medical centers. The study was consisted of four phases including 1) initial screening phase; 2) baseline phase; 3) double-blind treatment phase with single intrathecal injection of UCB-MNCs or sham procedure; and 4) follow-up phase. The trial protocol was explained in Supplement 1. The detailed description of methods was also published previously [17].

Males and females aged 4 to 14 years were eligible to enter the study if they were diagnosed with spastic CP based on standard criteria [18], gross motor function classification system (GMFCS) level 2 to 5, and white matter lesions in the brain imaging. The exclusion criteria were:

- Other types of CP (e.g. athetoid, ataxic, or mixed CP)
- Co-morbid neurological disorders
- History of malignancy, renal insufficiency, or liver failure
- Congenital infections (e.g. TORCH Syndrome)
- Severe anemia (hemoglobin < 8 mg/dl) or coagulation disorders
- Prior cell infusions

### Ethical issues

The final protocol was approved by the ethics committee of Tehran University of Medical Sciences (Number: IR.TUMS.VCRREC.1996.2506). The study was performed in accordance with the Declaration of Helsinki [19] and Good Clinical Practice guidelines. All information was explained to the parents of our participants and they were given a printed protocol of the study. It was explained that participation was optional and withdrawal was possible whenever they requested. The written informed consent was obtained from parents before the initiation of study procedures. We also explained the protocol to children and assent was achieved. The study was registered with Iranian registry of clinical trials; irct.ir (IRCT201706176907N13) on 12/07/2017 and ClinicalTrials.gov (NCT03795974) on 08/01/2019.

### Randomization and masking

The eligible cases were assigned in 4:1 ratio using permuted block randomization via interactive web response system to receive either UCB-MNCs or sham procedure; respectively. The blood of individuals assigned in UCB-MNC group was drawn and tested to figure out their HLA type. The results were compared to HLA types of umbilical cord blood cells. To provide better efficacy in the treatment and eliminate the risk of any related adverse event, subjects with 6/6 match at HLA-A, HLA-B, and HLA-DRB1 were treated with UCB-MNCs. Patient unique identification number and the assigned treatment code were achieved by a research associate in opaque envelopes. All participants, their parents, investigators, and the responsible statistician were masked during the study until the codes were broken at the end of the trial or if a severe adverse event occurred. Personnel staff responsible of cell preparations and HLA matching process was not blinded but they had no contacts with patients, parents, or investigators and no information about the clinical and imaging characteristics of participants was given to the unmasked staff.

### Cell preparation

The allogenic UCB-MNCs were obtained from umbilical cord blood units collected in Royan Cord Blood Bank. The written informed consent was obtained from healthy donors to use the umbilical cords for medical research purposes. Each frozen cord blood unit (-196 °C) was thawed at 37 °C and washed to reduce dimethyl sulfoxide concentration. The UCB-MNCs were isolated using 6% hydroxyethyl starch (HES) followed by LymphoprepTM (Stem cell Technology Inc., Canada) density gradient centrifugation. The cells were, then, suspended in animal product-free CO2-independent media and shipped to the hospitals at 15 °C.The basic characteristics of utilized UCBs are shown in Table 1. The UCB-MNCs were suspended in normal saline before the intrathecal injections.

**Table 1:** Characteristics of umbilical cord blood unit

**Cord Blood Unit**	Total nucleated cell count			
	Count × 10^6^	CFU × 10^5^	Viability	CD34^+^
			%	
**Pre-cryopreservation**	900 ± 305	NA	97.5 ± 2.3	0.04 ± 0.1
**Post-thaw**	791 ± 265	26.8 ± 14	90 ± 3.7	0.53 ± 0.3

*CFU* Colony forming units

### Intervention

The included cases were asked to lie down in lateral decubitus position with their knees drawn up to the chest. All participants were sedated to prevent awareness (masking) and to decrease the spasticity during the procedure. Lumbar puncture was performed in the HLA-matched individuals of experimental arm after washing the back with iodine. The 2 mL of the cerebrospinal fluid (CSF) were collected after the placement of spinal needle in the subarachnoid space and 1 mL was sent to the laboratory to determine the baseline CSF characteristics. A single dose of 5 × 10^6^ /kg body weight UCB-MNCs was, then, transplanted via intrathecal route slowly and the remained 1 mL CSF was infused at the last step.

The sham procedure included a small needle prick on the lower back skin of the sedated individuals in control group. The puncture site was covered in all cases. Participants were hospitalized in child neurology departments for 24 h to monitor the heart rates, temperature, blood pressure, and respiratory rates and to record early adverse events. No immunosuppressive agents were administered during the course of study.

### Assessments

Physical and neurological examinations were performed on participants in the initial screening phase. The GMFCS was used for the initial functional assessment. The screening tests were blood count (e.g. hemoglobin, white blood cells, and platelets), serum chemistry (e.g. liver function test, creatinine, and urea), prothrombin time, partial thromboplastin time, and electro-encephalography (EEG).

The gross motor function measure (GMFM)-66 and modified ashworth scale (MAS) were used to assess all cases at baseline, 1, 3, 6, and 12 months after the intervention. The pediatric evaluation of disability inventory (PEDI) and CP quality of life (CP-QoL) questionnaire were used to evaluate participants at baseline, 6, and 12 months after the intervention. The detailed description of clinical assessment tools was explained in Supplement 2.

The protocol of imaging was previously explained [17]. The magnetic resonance imaging (MRI) was performed on 1.5 T scanner (Philips Ingenia, Eindhoven, the Netherlands). The intravenous propofol (2 mg/kg dose) or thiopental (5 mg/kg dose) was used for sedation in cases that were not comfortable and moved during the imaging procedure to reduce motion artifacts. The MRI protocol for all participants was similar as followed: The 3D T1-weighted imaging (TR: 9.5 ms, TE: 4.6 ms, flip angle: 8°, FOV: 210 × 210 mm^2^, voxel size: 1 × 1 × 1 mm^3^) and 2D T2-weighted sequence (TR: 4000 ms, TE: 110 ms, flip angle: 90°, FOV: 230 × 230 mm^2^, voxel size: 0.8 × 0.8 × 3.5 mm^3^). The parameters of DTI included TR: 4228 ms, TE: 94 ms, flip angle: 90°, FOV: 224 × 224 mm^2^, and voxel size: 2.5 × 2.5 × 2.5 mm^3^.

DTI is an imaging technique that uses anisotropic diffusion to measure the microstructural changes of white matter pathways. The DTI post-processing was performed using ExploreDTI software [20] and included a cubic interpolation and robust estimation of tensors to correct subject motion, eddy current and EPI distortion. Non-rigid registration on the structural images was also performed. A whole-brain white matter tract construction was carried out for each participant using a linear interpolation. Seed point resolution was set at 1 mm × 1 mm × 1 mm with a seed fractional anisotropy threshold of 0.2 and an angle threshold of 50 degrees.

**Fig. 1:**
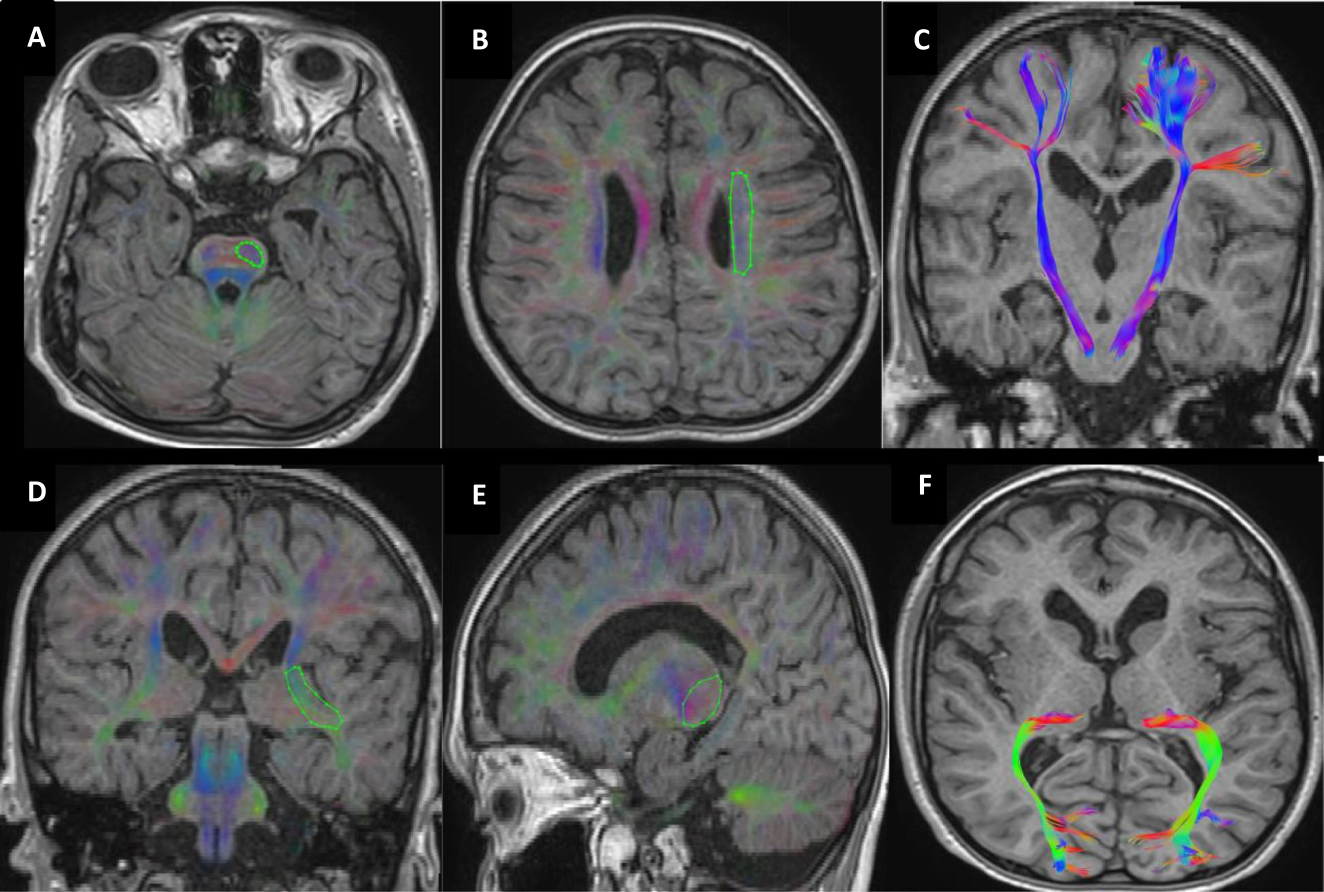
The ROI-based tractography. **A** (Pons) “AND” **B** (Centrum semi oval)  **C** (Corticospinal tract). **D** (Retro-lenticular of the internal capsule) “AND” **E** (Thalamus)  **F** (Posterior thalamic radiation)

Region of interest (ROI) based tractography was conducted. The predefined tracts including corticospinal tract (CST) and posterior thalamic radiation (PTR) were isolated in both hemispheres. For illustration of CST, the first ROI was drawn at the pons level and the second ROI was drawn at the centrum semi oval level using “AND” operation. The fibers of the middle cerebellar peduncle were excluded using “NOT” operation. For segmentation of PTR, the first ROI was drawn at retro-lenticular part of the internal capsule and the second ROI was at the thalamus using “AND” operation. All other tracts that not related to the PTR and were outside to ROIs were rejected by ROI “NOT” operation (Fig. 1). The mean value of fractional anisotropy (FA) and mean diffusivity (MD) were measured for each tract in both hemispheres. The two hemispheres of each participant were compared to each other and data of the most affected tracts were used in the analysis.

### Endpoints

The primary endpoints were the mean changes in the GMFM-66 scores from baseline to 12 months after intervention. The mean changes in the MAS, PEDI, and CP-QoL scores were also assessed. The secondary endpoints were the mean changes in FA and MD of the CST and PTR from baseline to 12 months after intervention.

Adverse events were recorded to assess the safety endpoint. All patients were monitored within 24 h after treatment. Furthermore, at each follow-up visit, the participants and their parents were requested to report any complication that was experienced. A phone number was also provided so adverse events could be reported in an easier way. Patients and their parents were also asked to visit the emergency department if any serious event occurred.

### Statistical analysis

The mean changes in GMFM-66 scores as the primary endpoints were used to estimate the sample size. It was calculated using repeated measures analysis of variance (ANOVA) by G*Power 3.1 software (University of Kiel, Germany). To achieve at least 80% power, the effect size of 0.25, two-sided α (the probability of type I error) of 0.05, and β (the probability of type II error) of 0.20 were considered and total sample size of 72 individuals (36 participants in each group) was estimated.

The protocol of analysis can also be found in [17]. The continuous variables were reported as means with standard deviation (SD) or standard error of the mean (SEM) and categorial variables were presented as percentages and were compared between groups using Pearson’s chi-squared test (gender, type of CP, and GMFCS). The distribution of variables was assessed using Kolmogrov-Smirnov. Two sided significance (*P*-value) lower than 0.05 showed the non-normal distribution (GMFM-66, PEDI, and CP-QoL) and higher than 0.05 showed the normal distribution of data (MAS and ROI-based data). The intention to treat approach was used and multiple imputation was conducted using Markov chain Monte Carlo to handle missing data. Generalized estimating equations (GEE) model was used to compare GMFM-66, MAS, PEDI, and CP-QoL mean scores between groups [21]. It was assumed that the interaction was between the intervention groups and time measurements. Exchangeable structure was considered for working correlation matrix and linear model was used. The model was adjusted to covariates including type of CP, GMFCS, gender, age, and weight of participants. Mann–Whitney U test was used to compare the primary endpoints between groups to further assess the clinical efficacy. Independent sample t-test was conducted to compare numeric variables in baseline and DTI data between groups. Statistical analyses were performed using the IBM SPSS Software, version 25.0 (SPSS Inc., Chicago, IL) and GraphPad Prism version 7.04. Two-sided significance testing was conducted, and *p*-values < 0.05 were considered statistically significant. Cohen’s d test with 95% confidence interval (CI) was used to measure the effect sizes that were classified as small (d: 0 to 0.20), medium (d: 0.20 to 0.50), and large (d > 0.50) using R statistical package (R Core Team, 2013).

## Results

### Patients

**Fig. 2:**
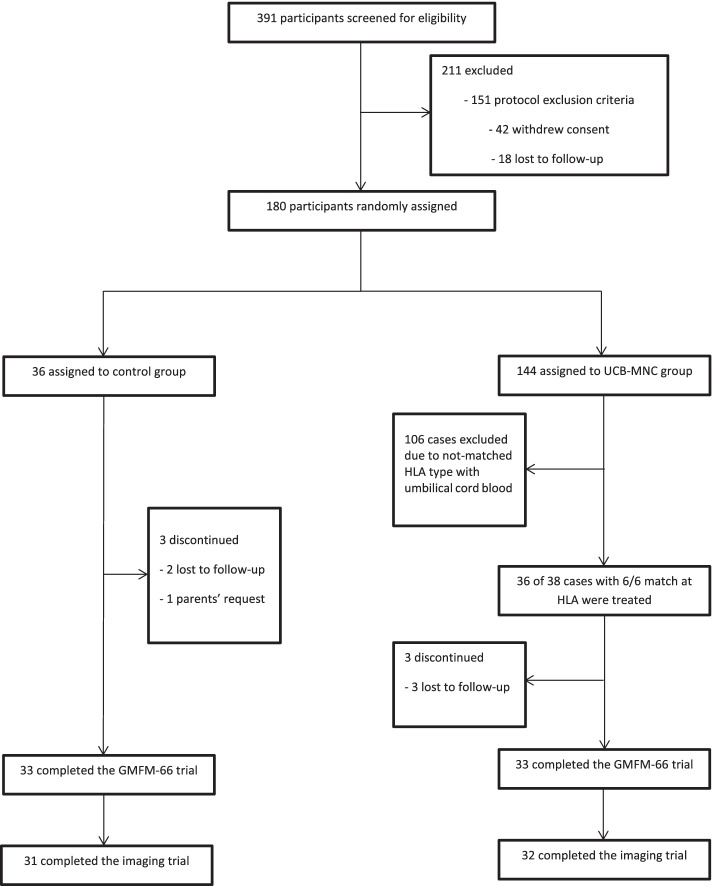
Flow chart of participants

The consolidated standards of reporting trials (CONSORT) were used in this study (Supplement 3). The initial screening phase began on July 23, 2017 and the first patient was allocated to study arm on August 19, 2017. The double-blind treatment phase lasted until November 24, 2018 and the study ended on December 2, 2019 after the last follow-up visit of the last participant. The primary screening was performed on 391 individuals to identify eligible cases and 180 patients were randomly assigned to study arms. Of 144 individuals assigned to the UCB-MNC group, 38 patients with 6/6 HLA match to umbilical cord blood were identified and 36 of them were randomly chosen and treated with UCB-MNCs. About 8% (six cases) discontinued the study. The reason for discontinuation were the lost to follow-up (*n* = 5—6.9%) or withdrawal of consent (*n* = 1—1.4%). Three participants (4.1%) were examined during the follow-up visits but the last DTI was not obtained due to the parents’ request (Fig. 2). The baseline demographic characteristics of each group are presented in Table 2.

**Table 2:** Baseline characteristic data

Variables	Control group	UCB-MNC group	*P*-value
Gender, n (%)			
Female	17 (47.2)	25 (69.45)	
Male	19(52.8)	11 (30.55)	0.11
Age (months)			
Mean ± SD	102.50 ± 29.91	112.51 ± 36.59	0.16
Weight (kg)			
Mean ± SD	17.32 ± 7.21	19.63 ± 8.51	0.19
Type of cerebral palsy, n (%)			
Spastic quadriplegia	32 (88.9)	29 (80.0)	
Spastic diplegia	4 (11.1)	7 (20.0)	0.30
GMFCS			
II/ III/ IV/V	4/4/11/17	6/5/11/14	0.25
GMFM-66			
Mean ± SD	66.30 ± 50.75	73.38 ± 49.15	0.45
MAS			
Mean ± SD	3.16 ± 0.97	2.91 ± 1.12	0.21
PEDI Self-care			
Mean ± SD	21.77 ± 16.15	27.58 ± 18.63	0.67
PEDI Mobility			
Mean ± SD	16.07 ± 14.01	22.11 ± 15.16	0.20
PEDI Social function			
Mean ± SD	26.63 ± 18.76	39.82 ± 18.42	0.67
CP-QOL			
Mean ± SD	369.8 ± 56.2	382.85 ± 63.02	0.36
FA corticospinal tract			
Mean ± SD	0.43 ± 0.06	0.45 ± 0.07	0.25
MD corticospinal tract^a^			
Mean ± SD	0.96 ± 0.07	0.94 ± 0.06	0.24
FA posterior thalamic radiation			
Mean ± SD	0.31 ± 0.04	0.35 ± 0.05	0.30
MD posterior thalamic radiation^a^			
Mean ± SD	1.06 ± 0.12	1.06 ± 0.08	0.98

*UCB-MNC* Umbilical cord blood mononuclear cell, *SD* Standard deviation, *GMFCS* Growth motor classification system, *GMFM* Growth motor function measurement, *PEDI* Pediatric evaluation of disability inventory, *CP-QOL* Cerebral palsy quality of life, *FA* Fractional anisotropy, *MD* Mean diffusivity

^a^The results should be divided by 1000

### Primary endpoints

**Fig. 3:**
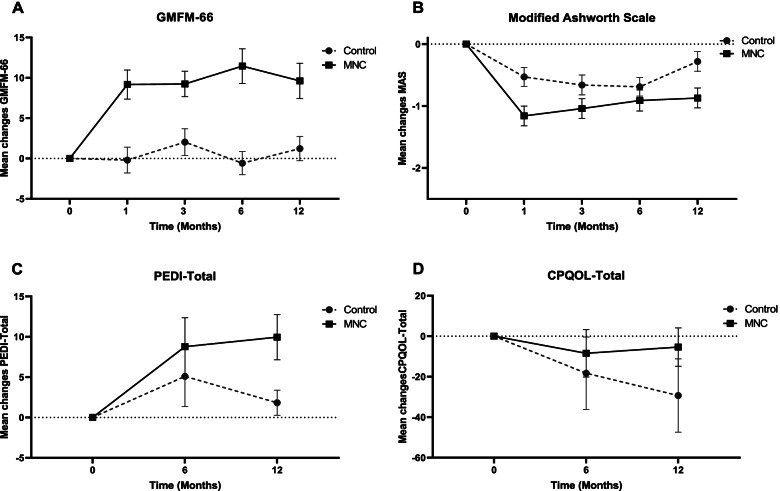
The changes in motor function (**A**), spasticity (**B**), disability (**C**), and quality of life (**D**) scores during the study period

There were 72 participants (36 cases in each group) who were included. The mean GMFM-66 scores, as the primary endpoints, were significantly higher in the UCB-MNC arm 12 months after treatment (9.62, 95%CI: 6.75 to 12.49) but not in control group (1.23, 95%CI: -3.33 to 5.80) (Fig. 3). The mean GMFM-66 score was statistically higher in the UCB-MNC arm compared to sham-control group with large effect size at the end of the study (β: 7.10, 95%CI: 2.08 to 12.76, Cohen’s d: 0.62) (Table 3 and 4). The mean MAS scores decreased significantly after 12 months of treatment (mean change: -0.87, 95%CI: -1.20 to -0.54) in the experimental group and significantly improved, compared to the control group with medium effect size (β: -0.58, 95%CI: -1.18 to -0.11, Cohen’s d: 0.36) (Table 3).

**Table 3:** The GMFM-66 and MAS mean difference within-groups (from baseline) and difference between groups

	**Test of within-group effects (mean change from baseline)**		**Test of between-groups effects (mean change from control group)**			
	**Control**	**MNC**	**MNC vs. Control**			
**Outcomes**	**Mean [95% CI]**	**Mean [95% CI]**	**β**	**95% CI**	**P.v**	**Cohen's d [95%CI]**
GMFM-66						
T1	-0.02 [-4.79, 4.75]	**9.24 [6.37, 12.11]**^a^	**9.34**	**[3.58, 13.09]**	**0.001**	0.87 [0.32, 1.41]
T2	2.03 [-2.97, 7.04]	**9.17 [6.42, 11.92]**	**7.03**	**[2.07, 11.24]**	**0.004**	0.73 [0.22, 1.23]
T3	-0.58 [-5.32, 4.15]	**11.26 [8.39,14.13]**	**11.91**	**[5.40, 18.37]**	** < 0.001**	0.94 [0.39, 1.48]
T4	1.23 [-3.33, 5.80]	**9.62 [6.75,12.49]**	**7.10**	**[2.08, 12.76]**	**0.006**	0.62 [0.07, 1.17]
MAS						
T1	**-0.53 [-0.87, -0.18]**	**-1.16 [-1.5, -0.85]**	**-0.62**	**[-1.01, -0.11]**	**0.014**	0.37 [0.14, 0.89]
T2	**-0.66[-0.99, -0.34]**	**-0.97 [-1.3, -0.66]**	-0.31	[-0.77, 0.10]	0.132	0.11 [-0.37, 0.60]
T3	**-0.69 [-1.03, -0.35]**	**-0.91 [-1.2,-0.58]**	-0.21	[-0.87, 0.21]	0.086	0.22 [-0.31, 0.75]
T4	-0.28 [-0.62, 0.05]	**-0.87 [-1.2, -0.54]**	**-0.58**	**[-1.18, -0.11]**	**0.016**	0.36 [0.20, 0.87]

^a^The bold font shows that results are statistically significant

*GMFM-66* Gross motor function measure-66, *MAS* Modified Ashworth scale, *T1* One-month data collection, *T2* Three-month data collection, *T3* Six-month data collection, *T4* One-year data collection

**Table 4:** The GMFM-66 mean difference within-group (from baseline) and difference between groups

GMFM-66	T_1_	T_2_	T_3_	T_4_
Control group	0.92 [-2.39,4.23]	2.25 [-1.02,5.52]	1.56 [-1.29,4.41]	3.50 [0.52,6.48]
Experimental group	9.17 [5.28,13.06]	9.24 [5.84,12.65]	11.24 [6.48,16.00]	10.48 [5.80,15.16]
*P*-value Between groups	0.009	0.007	0.003	0.04

*GMFM-66* Gross motor function measure-66, *T*_*1*_ One-month data collection, *T*_*2*_ Three-month data collection, *T*_*3*_ Six-month data collection, *T*_*4*_ One-year data collection

The mean PEDI scores increased significantly in all three dimensions in the UCB-MNC group after 12 months compared to the baseline but only mean of self-care scores significantly improved in the UCB-MNC arm compared to the control group after 12 months of treatment phase with large effect size (β: 3.56, 95%CI: 0.69 to 7.43, Cohen’s d: 0.73) (Table 5). The mean of scores of CP-QoL in two domains including “friends and family” and “participate in activities” was statistically higher experimental group compared to the control arm (Table 5).

**Table 5:** The PEDI and CP-QoL mean difference within-groups (from baseline) and difference between groups

	**Test of within-group effects mean change from baseline**			**Test of between-groups effects mean change**		
	**Control**	**MNC**		**MNC vs. control**		
**Outcomes**	**Mean [95% CI]**	**Mean [95% CI]**	**β**	**[95% CI]**	**P.v**	**Cohen's d [95% CI]**
PEDI Self-care						
T3	**1.89 [0.16, 3.62]**	**3.63 [2.21, 5.04]**^a^	1.74	[-1.48, 4.96]	0.290	0.28 [-0.20, 0.77]
T4	0.15 [-1.63, 1.95]	**3.79 [2.30, 5.28]**	**3.56**	**[0.69, 7.43]**	**0.018**	0.73 [0.19, 1.26]
PEDI Mobility						
T3	0.13 [-1.69, 1.91]	**2.57 [1.44, 3.69]**	2.34	[-0.07, 5.02]	0.05	0.56 [-0.01, 1.10]
T4	0.81 [-1.09, 2.72]	**1.83 [0.65, 3.01]**	1.52	[-1.14, 4.18]	0.31	0.62 [-0.25, 0.88]
PEDI Social function						
T3	**3.04 [0.86, 5.23]**	2.58 [-0.11, 5.27]	-0.44	[-4.14,3.26]	0.81	0.07 [-0.56, 0.41]
T4	1.35 [-0.93, 3.60]	**4.37 [1.53, 7.20]**	3.09	[-0.76,6.96]	0.11	0.56 [-0.02, 1.08]
PEDI Total						
T3	**5.09 [1.35, 8.83]**	**8.77 [4.76, 12.78]**	3.73	[-4.07, 11.54]	0.348	0.40 [-0.13, 0.94]
T4	1.58 [-2.30, 5.46]	**9.95 [5.73, 14.17]**	**8.59**	**[0.44, 16.75]**	**0.039**	0.78 [0.19, 1.37]
CPQoL						
Friends and family						
T3	-1.78 [-8.78,5.20]	3.21 [-3.16, 9.58]	5.24	[-3.71, 14.19]	0.251	0.24[-0.27, 0.75]
T4	-10.36 [-17.45,-3.28]	-1.32 [-7.54, 4.88]	**9.10**	**[0.20, 17.99]**	**0.045**	0.63[0.10, 1.16]
Participate in activities						
T3	-2.20 [-4.84, 0.43]	-1.62 [-6.25, -1.60]	0.59	[-3.14, 4.34]	0.755	0.10[-0.40,0.62]
T4	-4.20 [-6.88,-1.53]	-2.23 [-4.63, 0.17]	**3.93**	**[0.21, 7.65]**	**0.038**	0.58[0.01,0.99]
Communication						
T3	-2.65 [-4.42,-0.88]	-3.92 [-2.95, 0.59]	-1.26	[-4.17, 1.64]	0.394	0.26[-0.75,0.22]
T4	-1.99 [-3.83,-0.15]	0.56 [-1.29, 2.43]	2.50	[-0.06, 5.17]	0.90	0.02 [-0.54,0.49]
Physical health						
T3	-1.27 [-6.78,4.29]	0.42 [-8.24, 9.60]	2.52	[-7.75, 12.79]	0.630	0.07[-0.440.58]
T4	-3.54[-9.12,2.02]	5.93 [-3.02, 14.88]	9.56	[-0.64, 19.77]	0.06	0.53 [0.01,1.05]
Special equipment						
T3	-0.74 [-2.95,1.42]	0.18 [-2.11, 2.47]	0.85	[-2.30, 4.0]	0.597	0.08 [-0.43,0.59]
T4	0.45 [-1.76, 2.67]	0.66 [-1.57, 2.90]	0.05	[-3.08, 3.19]	0.971	0.03[-0.47,0.54]
Pain and impact of disability						
T3	-0.86 [-6.33,4.59]	-3.04 [-7.48, 1.40]	-2.41	[-9.27,4.46]	0.491	0.14 [-0.65,0.37]
T4	1.31 [-4.22,6.58]	2.69 [-1.63, 7.02]	1.37	[-5.46, 8.20]	0.494	0.09[-0.41,0.60]
Access to Services						
T3	-6.09 [-11.43,-0.74]	-2.66 [-9.52, 0.89]	3.71	[-4.45, 11.89]	0.372	0.22[-0.28,0.75]
T4	-8.66 [-14.08,-3.24]	-5.28 [-11.42, 0.84]	3.39	[-4.72, 11.51]	0.412	0.19[-0.31,0.70]
Family Health						
T3	-3.28 [-6.21,-0.35]	-3.47 [-5.88, -1.07]	-0.10	[-3.80, 3.59]	0.956	0.01 [-0.51,0.51]
T4	-2.52 [-5.49,0.44]	-4.88 [-7.22, -2.54]	-2.42	[-6.10, 1.25]	0.196	0.24 [-0.76,0.26]
CPQoL-total						
T3	-18.3 [-36.19,-0.37]	-8.42[-28.27, 11.41]	10.03	[-14.91, 34.97]	0.431	0.16[0.35, 0.67]
sT4	-29.3 [-47.5, -11.2]	-5.33 [-24.66, 14.0]	24.08	[-0.70, 48.86]	0.057	0.46[-0.05, 0.98]

^a^The bold font shows that results are statistically significant

*CP-QoL* Cerebral palsy quality of life child, *PEDI* Pediatric evaluation of disability inventory, *T3* Six-month data collection, *T4* One-year data collection

### Secondary endpoints

**Fig. 4:**
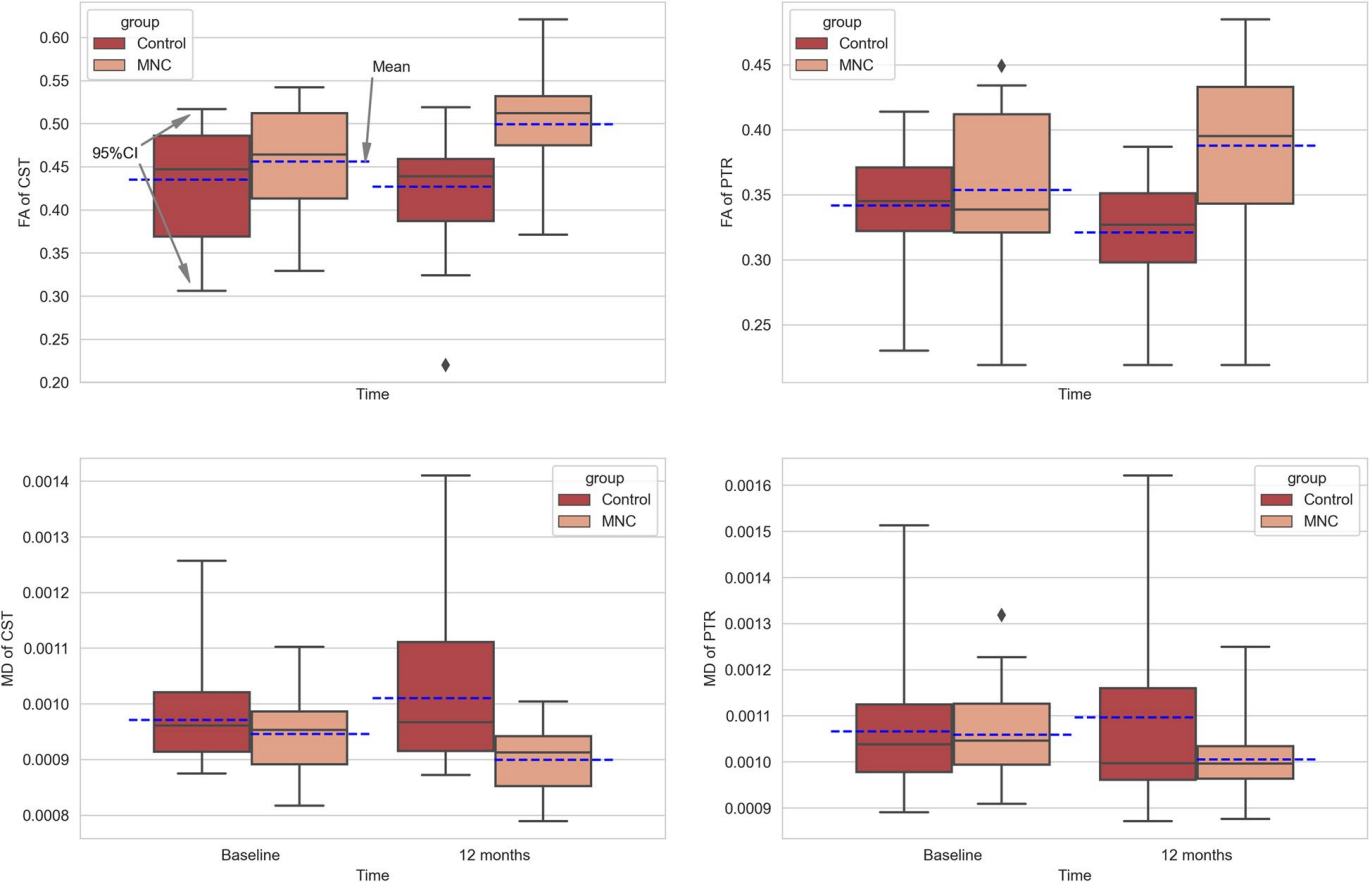
Box plot to compare the fractional anisotropy and mean diffusivity of corticospinal tract and posterior thalamic radiation within and between groups

The DTI data indicated that mean FA increased significantly in the experimental arm in 12 months after intrathecal cell injections (CST mean change: + 0.042, 95%CI: 0.03 to 0.04; PTR mean change: + 0.032, 95%CI: 0.02 to 0.03) and was statistically higher than control group with large effect size (CST Cohen’s d: 0.99 and PTR Cohen’s d: 1.05) (Table 6) (Fig. 4). The mean MD decreased significantly in the UCB-MNC group after 12 months of intervention (CST mean change: -0.044 × 10^–3^, 95%CI: -0.04 × 10^–3^ to -0.03 × 10^–3^; PTR mean change: -0.050 × 10^–3^, 95%CI: -0.05 × 10^–3^ to -0.04 × 10^–3^) and was statistically lower than control group with large effect size (CST Cohen’s d: 0.95 and PTR Cohen’s d: 0.57) (Table 6) (Fig. 3). The adverse events are presented in Table 7. Total number of 30 adverse events were observed among 17 cases who received UCB-MNCs during 24 h after treatment. No serious events were reported and they were resolved without causing any complications. During the follow-up visits no adverse events were reported by the patients or their parents that showed that the short-term adverse events were related to the lumbar puncture.

**Table 6:** Secondary endpoints analysis

Outcome	Control (*n* = 36)	UCB-MNC (*n* = 36)
**Fractional anisotropy**		
**Corticospinal tract**		
12 months mean (SD)	0.42 (0.06)	0.49 (0.06)
Mean changes from baseline (95% CI)	-0.007 (-0.010 to -0.002)	0.042 (0.03 to 0.04)
Difference vs control (95% CI)	-	0.069 (0.03 to 0.10)
Cohen’s d (95%CI)	-	0.99 (0.56 to 1.42)
**Posterior thalamic radiate**		
12 months mean (SD)	0.32 (0.04)	0.38 (0.05)
Mean chnges from baseline (95% CI)	-0.020 (-0.02 to -0.01)	0.032 (0.02 to 0.03)
Difference vs control (95% CI)	-	0.064 (0.3 to 0.9)
Cohen’s d (95%CI)	-	1.05 (0.62 to 1.47)
**Mean diffusivity**^a^		
**Corticospinal tract**		
12 months mean (SD)	1.00 (0.12)	0.90 (0.06)
Mean changes from baseline (95% CI)	0.040 (0.02 to 0.05)	-0.044 (-0.04 to -0.03)
Difference vs control (95% CI)	-	-0.10 (-0.15 to -0.05)
Cohen’s d (95%CI)	-	0.95 (0.49 to 1.41)
**Posterior thalamic radiate**		
12 months mean (SD)	1.09 (0.19)	1.01 (0.08)
Mean chnges from baseline (95% CI)	0.033 (0.01 to 0.05)	-0.050 (-0.07 to -0.04)
Difference vs control (95% CI)	-	-0.09 (-0.16 to -0.01)
Cohen’s d (95%CI)	-	0.57 (0.06 to 1.07)

^a^The results should be divided by 1000

**Table 7:** Number of participants with adverse events

Event	Control group	UCB-MNC group
Fever	0	2
Irritability	3	7
Headaches	1	9
Low back pain	0	11
Vomiting	0	1

## Discussion

There are several factors that can cause CP but the underlying pathophysiological mechanisms are common. The elevated levels of pro-inflammatory cytokines, oxidative stress, and deprivation of growth factors are observed in this condition that can lead to myelination defects and gliosis as well as thalamic and cortical damages [22]. The microglial cells were found to play a key role in neuro-toxicity after the brain injury. The plausible underlying mechanisms for the therapeutic effectiveness of stem cells include immunomodulation as well as secretion of trophic factors, anti-oxidant molecules, angiogenic, anti-inflammatory, anti-fibrotic, and anti-apoptotic agents that can enhance the repair of injured tissue [23–26]. The stem cells are capable to migrate to the site of injury (homing) and differentiate into new cells but this mechanism of action was reported to have limited efficacy [27].

The current study showed that the gross motor function, muscle tone, and disability score improved in participants treated with the single dose of UCB-MNCs compared to cases in the control group over one year after the treatment. The improvements in motor function and spasticity in the experimental group reduced in the last follow-up visit (12 months) compared to the previous one (6 months). This may show the temporary effects of cell therapy that can be resolved with repeated cell injections in different time periods.

The gross motor function was found to be increased and the spasticity decreased after one month of cell injection that continued until the end of follow-up visits. The self-care, mobility, and social function of individuals treated with UCB-MNCs were also increased but most domains of quality of life did not show significant improvements at the end of the trial. The MNCs can be transplanted via different routes and isolated from different sources. Four prior placebo-controlled studies reported that the intravenous/intra-arterial injection of UCB-MNCs was associated with significant improvements in motor function [14, 15, 28, 29]. A prior placebo-controlled double-blind study reported that intravenous infusion of UCB-MNCs was effective and cases administered higher matched units (HLA full-matched or 1 mismatched) had greater improvements in gross motor function than those administered with the HLA 2 mismatched units [15]. Our study showed that intrathecal injection of lower dose cord blood cells (5 × 10^6^/kg vs. 3 × 10^7^/kg) can also be effective in patients with HLA full-matched. The autologous bone marrow MNC (BM-MNC) transplantation was also shown to be safe and effective in CP [30–34]. The results of some studies were, however, partially inconsistent with our findings. A recent clinical trial indicated that children with CP who were treated with BM-MNCs had significant improvements in gross motor function compared to the baseline but no changes were observed after comparing to the control group [35]. The discrepancies could be due to methodological differences including cell dose (four times at 1 × 10^6^/ kg [34] vs. 5 × 10^6^/ kg in the present study), cell preparation, or statistical analysis. Another trial demonstrated that BM-MNC injection markedly increased the quality of lives of individuals with CP [30] that was not found in our trial. The difference might be due to information bias as the data cannot be objectively evaluated and should be further assessed in future studies.

The DTI data of the experimental group showed that the mean FA in CST and PTR increased and the mean MD reduced in these tracts one year after the treatment; compared to the baseline and control group. The increments in FA changes after UCB-MNCs intravenous infusion were also reported in two prior studies, in the CST, spinothalamic tract, and anterior thalamic radiation [14, 15]. Other assessments including ^18^F-fluorodeoxyglucose positron emission tomography (PET) and EEG were conducted in few prior studies and reported improved inflammation in posterior white matter [14, 28] and reduced average delta/alpha band power ratio in posterior cerebral cortex [15]; respectively. These data can objectively show the efficacy of UCB-MNC treatment in people with CP and imply the improvements in CNS structure.

This trial had different strengths. To our knowledge, this is one of the few studies that used quantitative DTI to assess the efficacy of stem cell injection in children and adolescents with CP. Randomization and blinding were other strengths of the clinical trial that can reduce the measurement and observer-expectancy bias. The multi-center prospective population-based design of the study enhanced the external validity of the results. There were also some limitations to our study. The single dose of UCB-MNC was administered in our participants. Repeated injections might increase the efficacy of the cells. Small sample size was another limitation that should be resolved in future trials. Although prior analyses reported better efficacy of UCB-MNCs in fully HLA-matched or 1-mismatched patients compared to cases with 2-mismatched [15], studies reported that transplanting unmatched or partially matched UCB-MNCs were safe in cases with CP [36–38]. This can significantly reduce the time needed to do the procedures and provide treatments to more patients. There are various other types of stem cells that were used in CP including MSCs, neural progenitor cells, embryonic tissue, and olfactory ensheathing cells [9–11, 39–42]. The efficacy of different cells should be compared to each other and the optimal dose and route of administration should be determined in future studies to find the most appropriate protocol of cell transplantation. Longer follow-up periods are suggested to realize the long-term safety and efficacy of stem cells. Other imaging data including magnetic resonance spectroscopy can also be helpful to better understand the effects of stem cells on neuronal repair.

## Conclusion

The gross motor, muscle tone, and functional abilities as well as white matter structure of brain in children and adolescents with CP significantly improved with intrathecal injection of UCB-MNCs.

## Supplementary Information

**Additional file 1.**

**Additional file 2.**

**Additional file 3.**

## Data Availability

The datasets generated and/or analysed during the current study are not publicly available but are available from the corresponding author on reasonable request.

## Abbreviations

ANOVA: Analysis of variance
BM-MNC: Bone-marrow mono-nuclear cell
CI: Confidence interval
CP: Cerebral palsy
CP-QoL: Cerebral palsy quality of life
CST: Corticospinal tract
DTI: Diffusion tensor imaging
EEG: Electro-encephalography
FA: Fractional anisotropy
GEE: Generalized estimating equations
GMFCS: Gross motor function classification system
GMFM: Gross motor function measure
MD: Mean diffusivity
MAS: Modified Ashworth scale
MRI: Magnetic resonance imaging
PEDI: Pediatric evaluation of disability inventory
PET: Positron emission tomography
PTR: Posterior thalamic radiation
ROI: Region of interest
SD: Standard deviation
SEM: Standard error of the mean
UCB-MNC: Umbilical cord blood mono-nuclear cell
