# Supplementary material for: The safety and efficacy of umbilical cord blood mononuclear cells in individuals with spastic cerebral palsy: a randomized double-blind sham-controlled clinical trial
Source: BMC Neurol. 2022 Mar 29;22:123. doi: 10.1186/s12883-022-02636-y (PMC8966246; doi:10.1186/s12883-022-02636-y)
Supplement: Supplementary file 1 — Additional file 1. [file 12883_2022_2636_MOESM1_ESM.docx]

# Background Cerebral palsy (CP) is consisted of a group of developmental disabilities in the field of motor function and is one of the major problems of pediatric neurology. Currently, there are no standard curative medical or surgical treatments for CP. Stem cell therapy is one of a new and hopeful therapeutic methods of therapy for different neurological conditions. Stem cells are defined as pluripotent cells with the ability of self-renewal and the capacity of differentiation into the other cell types. The release of neurotrophic factors, anti-oxidant molecules, angiogenic, anti-inflammatory, anti-fibrotic, and anti-apoptotic agents after stem cell transplantation can improve the tissue injury. Furthermore, the capacity of these cells to regenerate and differentiate to new cells is another proposed mechanism of action to repair damaged neurons.

# Aim

This multi-center population-based randomized double-blind trial will assess the efficacy and safety of umbilical cord blood mononuclear cells (UCB-MNCs) in the treatment of CP, compared to the sham-procedure.

# Design

A randomized double-blind sham-controlled trial will be conducted. Children aged 4-14 years who meet the diagnostic criteria for spastic CP will be enrolled. They have to have gross motor function classification system (GMFCS) of level 2 to 5, and white matter lesions in the brain imaging. Patients will be excluded if they have other types of CP (e.g. athetoid, ataxic, or mixed CP) or history of co-morbid neurological conditions (e.g. epilepsy), malignancy, or renal failure. Congenital infections (e.g. TORCH), severe anemia, coagulation disorders, and prior cell infusion are other exclusion criteria.

# Randomization

Eligible participants will be randomly assigned in a 4:1 ratio by permuted block randomization via an interactive web response system. The responsible statistician will not be involved in study conduct or monitoring. The blood of individuals who will be assigned in UCB-MNC group will be drawn and tested to figure out their human leukocyte antigen (HLA) type. The results will be compared to HLA types of umbilical cord blood cells. Subjects with 6/6 match at HLA-A, HLA-B, and HLA-DRB1 will be treated with UCB-MNCs.

# Blinding

Personnel staff responsible of cell preparations and HLA matching process will not be blinded but they won’t have contacts with patients, parents, or investigators and no information about the clinical and imaging characteristics of participants will be given to the unmasked staff. All participants, their parents, investigators, and the responsible statistician were masked during the study. All participants will be sedated to prevent awareness.

# cell preparation and injection

## The allogenic UCB-MNCs are obtained from umbilical cord blood units collected in Royan Cord Blood Bank. The donors are selected from full-term healthy mothers who had normal vaginal delivery without complication. The blood samples of donors will be collected and tested for reactive transmissible infectious agents including human immunodeficiency virus, hepatitis B virus, hepatitis C virus, and cytomegalovirus. Each frozen cord blood unit (-196°C) will be thawed at 37°C and washed to reduce dimethyl sulfoxide concentration. The UCB-MNCs will be isolated using 6% hydroxyethyl starch (HES) followed by LymphoprepTM (Stem cell Technology Inc., Canada) density gradient centrifugation. The cells will, then, suspended in animal product-free CO2-independent media and shipped to the hospitals at 15°C.

## All included cases will be asked to lie down in lateral decubitus position with their knees drawn up to the chest. A single dose of 5 x 10^6^ /kg body weight UCB-MNCs will be transplanted via intrathecal route within 2 minutes in the experimental group. The sham procedure will be a small needle prick on the lower back skin of the sedated individuals in control group. The puncture site will be covered in all cases.

# Endpoints

The primary endpoints will be the mean changes in the gross motor function measure (GMFM)-66, modified ashworth scale (MAS), pediatric evaluation of disability inventory (PEDI), and CP quality of life (CP-QoL) scores from baseline to 12 months after intervention. The quantitative diffusion tensor imaging (DTI) will also be performed to assess the alteration in white matter integrity. The secondary endpoints will be the mean changes in fractional anisotropy (FA) and mean diffusivity (MD) of corticospinal tract (CST) and posterior thalamic radiate (PTR) from baseline to 12 months after intervention. Adverse events were recorded to assess the safety endpoint.

# Sample Size

The mean changes in GMFM-66 scores were used to estimate the sample size. It was calculated using repeated measures analysis of variance (ANOVA) by G*Power 3.1 software (University of Kiel, Germany). To achieve at least 80% power, the effect size of 0.25, two-sided α (the probability of type I error) of 0.05, and β (the probability of type II error) of 0.20 were considered and total sample size of 72 individuals (36 participants in each group) was estimated.

# Statistical Analysis

Statistical analysis will be performed by the statistician who is masked to the study arms. Continuous variables will be reported as means and standard error mean, 95% confidence interval (CI) or standard deviation. Categorical variables will be described using percentage. Analyses of between-group differences will be performed according to the intention to treat (ITT) approach. All randomized patients who will receive study drug or sham procedure will be included. Missing data will be handled by multiple imputation.

Generalized estimating equations (GEE) model will be used to compare GMFM-66, MAS, PEDI, and CP-QoL mean scores between groups. It will assume that the interaction will be between the intervention groups and time measurements. Exchangeable structure will be considered for working correlation matrix and linear model will be used. The model will be adjusted to covariates including type of CP, GMFCS, gender, age, and weight of participants. Independent sample t-test will be conducted to compare numeric variables in baseline and DTI data between groups.

# Ethics

## The ethics committee of the Tehran University of Medical Sciences approved the final protocol of the study (Number: IR.TUMS.VCRREC.1996.2506). The study will be performed in accordance with the Declaration of Helsinki and Good Clinical Practice guidelines. All information will be explained to the parents of our participants and they will be given a printed protocol of the study. It will be explained that participation is optional and withdrawal is possible whenever they request. The written informed consent will be obtained from parents before the initiation of study procedures. We also will explain the protocol to children and assent will be achieved. The study is registered with Iranian Registry of Clinical Trials; IRCT.ir, number IRCT201706176907N13 on 12/07/2017 and ClinicalTrials.gov (NCT03795974) on 08/01/2019.

# Sponsor

Tehran University of Medical Sciences

# Date trial started

July 23, 2017
