# Supplementary material for: The safety and efficacy of umbilical cord blood mononuclear cells in individuals with spastic cerebral palsy: a randomized double-blind sham-controlled clinical trial
Source: BMC Neurol. 2022 Mar 29;22:123. doi: 10.1186/s12883-022-02636-y (PMC8966246; doi:10.1186/s12883-022-02636-y)
Supplement: Supplementary file 2 — Additional file 2. [file 12883_2022_2636_MOESM2_ESM.docx]

**Gross motor function measure (GMFM)-66**

The GMFM-66 was found through Rasch analysis to best describe the gross motor function of children with CP of varying abilities and is a 66 item subset of the original 88 items (1). It has a unidimensional scale providing interval scaling rather than the ordinal scaling of the GMFM-88. It was shown that inter-rater reliability of Farsi version of this scale for all dimensions was 0.97 to 0.99 and the intra-rater reliability was 0.99 (2). Cronbach's alpha coefficient for all dimensions was 0.78 to 0.94 (2).

**Modified ashworth scale (MAS)**

The scale was designed to assess the muscle tone:

| **0**: No increase in muscle tone (normal) |
| --- |
| **1**: Slight increase in muscle tone, manifested by a catch and release or by minimal resistance at the end of the range of motion when the affected part(s) is moved in flexion or extension |
| **1+ (2)**: Increase in muscle tone, manifested by a catch, followed by minimal resistance throughout the remainder (less than half) of the range of motion |
| **3**: More marked increase in muscle tone through most of the range of motion, but affected part(s) easily moved |
| **4**: Considerable increase in muscle tone, passive movement difficult |
| **5**: Affected part(s) rigid in flexion or extension |

Elbow flexor, wrist flexor, knee extensor, hip adductor, and ankle plantar flexor were examined on the spastic side(s) and the mean MAS score was recorded in each individual. Participants were in sitting position to examine hip adductor and supine position to test other muscles.

**The pediatric evaluation of disability inventory (PEDI)**

It was developed to assess the performances of children with disabilities in 3 dimensions including self-care, mobility, and social function. The Farsi version of PEDI in children with CP was reported to have high internal consistency (Cronbach's alpha: 0.94 to 0.98). The results of test-retest reliability were excellent in self-care (0.99) and social performance (1); and good in mobility dimension (0.66) (3).

**Cerebral palsy quality of life (CP-QoL)**

The questionnaire was developed to evaluate the well-being across different domains of life in children and adolescents with CP. The CP-QoL-child form with primary caregiver proxy report was used in this trial. The domains included family and friends, participation in activities, communication, physical health, special equipment, pain and bother, access to services, and family health. Good internal consistency (Cronbach's alpha: 0.61 to 0.87) and moderate to good test-retest reliability (0.47 to 0.84) in all domains were reprted in the Farsi version of questionnaire (4).

**References**

1. Russell DJ, Rosenbaum PL, Wright M, Avery LM. Gross Motor Function Measure (GMFM-66 & GMFM-88) User's Manual. 2013. London: Mac Keith Press.
2. Salehi R, Keshavarz A, Negahban H, Saeedi A, Shiravi A, Ghorbani S, Taghizade G, Azizi R. Development of the Persian version of gross motor function measure-88 (GMFM-88): A study of reliability. Trends in Medical Research. 2015;10(3):69-74.
3. Moradi Abbasabadi M, Akbarfahimi N, Hosseini SA, Rezasoltani P. Reliability of the Persian Version of the pediatric evaluation of disability inventory in 3 to 9-year old children with cerebral palsy. Journal of Mazandaran University of Medical Sciences. 2015;25(130):129-37.
4. Soleimani F, Vameghi R, Kazemnejad A, Fahimi NA, Nobakht Z, Rassafiani M. Psychometric properties of the persian version of cerebral palsy quality of life questionnaire for children. Iranian journal of child neurology. 2015;9(1):76-86.
